# The Italian external quality assessment for *RAS* testing in colorectal carcinoma identifies methods-related inter-laboratory differences

**DOI:** 10.1186/s12967-015-0655-1

**Published:** 2015-09-03

**Authors:** Nicola Normanno, Carmine Pinto, Francesca Castiglione, Francesca Fenizia, Massimo Barberis, Antonio Marchetti, Gabriella Fontanini, Gaetano De Rosa, Gian Luigi Taddei

**Affiliations:** Cell Biology and Biotherapy Unit, Istituto Nazionale per lo Studio e la Cura dei Tumori “Fondazione Giovanni Pascale”-IRCCS, Via M. Semmola, 80131 Naples, Italy; Laboratory of Pharmacogenomics, Centro di Ricerche Oncologiche di Mercogliano (CROM), Istituto Nazionale Tumori “Fondazione Giovanni Pascale” IRCCS, Naples, Italy; Medical Oncology, S. Maria Hospital - IRCCS, Reggio Emilia, Italy; Department of Human Pathology and Oncology, University of Florence, Florence, Italy; Department of Pathology and Laboratory Medicine, European Institute of Oncology, Milan, Italy; Center of Predictive Molecular Medicine, University-Foundation, CeSI Biotech, Chieti, Italy; Division of Pathological Anatomy, Department of Surgical, Medical, Molecular Pathology and Critical Care, University of Pisa, Pisa, Italy; Pathology Section, Department of Advanced Biomedical Sciences, University of Naples Federico II, Naples, Italy

**Keywords:** Colorectal cancer, Quality assurance, RAS mutations

## Abstract

**Background:**

In 2014 the European Medicines Agency included exon 2, 3 and 4 *KRAS* and *NRAS* testing for the selection of metastatic colorectal cancer (mCRC) patients eligible for the therapy with anti-EGFR monoclonal antibodies. The Italian Association of Medical Oncology (AIOM) and the Italian Society of Pathology and Cytology (SIAPEC) organized an external quality assessment (EQA) scheme for CRC to evaluate inter-laboratory consistency and to ensure standardization of the results in the transition from *KRAS* to all-*RAS* testing.

**Methods:**

Ten formalin fixed paraffin embedded specimens including *KRAS/NRAS* (exons 2, 3, 4) and *BRAF* (codon 600) mutations were validated by three referral laboratories and sent to 88 participant centers. Molecular pathology sample reports were also requested to each laboratory. A board of assessors from AIOM and SIAPEC evaluated the results according to a predefined scoring system. The scheme was composed of two rounds.

**Results:**

In the first round 36 % of the 88 participants failed, with 23 centers having at least one false positive or false negative while 9 centers did not meet the deadline. The genotyping error rate was higher when Sanger sequencing was employed for testing as compared with pyrosequencing (3 vs 1.3 %; p = 0.01; Pearson Chi Square test). In the second round, the laboratories improved their performance, with 23/32 laboratories passing the round. Overall, 79/88 participants passed the RAS EQA scheme. Standardized Human Genome Variation Society nomenclature was incorrectly used to describe the mutations identified and relevant variations were noticed in the genotype specification.

**Conclusion:**

The results of the Italian RAS EQA scheme indicate that the mutational analyses are performed with good quality in many Italian centers, although significant differences in the methods used were highlighted. The relatively high number of centers failing the first round underlines the fundamental role in continued education covered by EQA schemes.

## Background

Cetuximab and panitumumab are two monoclonal antibodies (mAbs) that bind the extracellular domain of the EGFR, block its interaction with ligands and inhibit its downstream signalling [1, 2]. These drugs were initially proved to be effective only in a subgroup of metastatic colorectal cancer (mCRC) carrying a wild-type *KRAS* gene in codons 12 and 13 of exon 2 [3–5] and in 2009 received the European Medicines Agency (EMA) approval for this molecularly selected group of mCRC patients. Exon 2 *KRAS* mutations are usually detected in approximately 30–40 % of CRC patients, representing up to 90 % of all *KRAS* mutations [5]. More recently, less frequent mutations in exon 3 and 4 of *KRAS* and mutations in exons 2, 3 and 4 in *NRAS* have been demonstrated to be equally associated with resistance to EGFR monoclonal antibodies [6–8]. Although *RAS* mutations are an early event in colon tumorigenesis, heterogeneous expression of *RAS* variants in CRC has been recently reported [9].

On the basis of these studies, in 2014 the EMA restricted the use of cetuximab and panitumumab to patients with *RAS* (exon 2, 3 and 4 of *KRAS* and *NRAS* genes) wild-type status. Following this updating, *KRAS* and *NRAS* testing has become a fundamental part in the decision-making process for the identification of the correct pharmacological approach to mCRC. Other genes involved in EGFR downstream pathways, such as *BRAF*, *PIK3CA* and *PTEN*, might be involved in the development of resistance mechanisms to cetuximab and panitumumab in CRC, although their role is not completely clarified [10–12]. Despite this controversial position, the analysis of the mutational status of *BRAF* is often required by the oncologists, since it is a strong negative prognostic biomarker for mCRC patients and *BRAF* mutant patients are often treated with aggressive therapeutic regimens.

*RAS* and *BRAF* molecular tests must produce accurate, reliable and readily available results, since they directly influence treatment decisions. Up to date, many different testing methods exist and no further indications have been provided by the EMA on which test is the most reliable. Moreover, the complexity of the analyses required is increasing with the identification of new predictive biomarkers and the development of new precision drugs. For all these reasons, today the External Quality Assessment (EQA) of the laboratories performing these analyses is considered an essential step to ensure inter-laboratory consistency and to obtain the harmonization and standardization of the results. In 2013, AIOM and SIAPEC-IAP organized the EQA scheme for CRC including the analysis of *BRAF* exon 15 mutations in codon 600, in addition to exon 2, 3 and 4 of *KRAS* and *NRAS* genes. This article summarizes the results obtained during the scheme and evaluates shortcomings in both genotyping and clinical reports risen in the transition from *KRAS* to all-*RAS* testing.

## Methods

### Validation of control samples

Formalin fixed paraffin embedded (FFPE) specimens derived from resected mCRC were collected at referral surgical pathology departments. One 10-µm-thick slide derived from samples with an adequate content of tumor cells (≥50 %) was selected and analysed by three referral centers that were selected based on their experience in molecular pathology, their track of scientific publications and their expertise in EQA organization: the Center of Predictive Molecular Medicine at CeSI Biotech in Chieti, the Department of Human Pathology and Oncology at the University of Florence and the Laboratory of Pharmacogenomics, at CROM—INT “Fondazione Giovanni Pascale” in Mercogliano. Three different methods in a blinded fashion were used (pyrosequencing, Ion AmpliSeq™ Colon and Lung Cancer panel—Thermo Fisher Scientific©, Sanger sequencing). Among the samples analysed, 20 were chosen for which a 100 % concordance between the referral centers was obtained and a percentage of mutant allele >15 % was observed. The percentage of mutant allele in each sample used in first and in second round was quantified using the Ion Torrent™ panel. For each sample, the thirtieth, the sixtieth and the last section obtained from the block were reanalysed for *KRAS*, *NRAS* and *BRAF* mutational status by using pyrosequencing, to ensure that the mutation was homogeneously present within the block.

The same samples were sent to all participating laboratories. Due to the high number of participants, the scheme focused on the mutational analysis only, because it was not feasible to provide sections for pathological review that will be addressed in a dedicated program.

### Registration of the participants and shipment of the samples

Laboratories that performed the analyses of all the three genes were invited to participate to the EQA. The centers could register at the http://www.rasquality.it web site and were asked to extract DNA and perform the analyses by using their routine methods. In each round, one slide of 10-µm thickness for each of the 10 samples was sent to the laboratories which correctly registered on the web site. Each specimen was given a random code automatically created by an application of the web site, to avoid exchange of information among the centers and to univocally identify the samples. The deadline for inserting the results on the web site was set 3 weeks after the shipment date. The laboratories were also asked to provide information on the methods used to perform the mutational analyses. A molecular pathology sample report, uploaded and sent together with the results, was requested to each laboratory and was used only for a qualitative evaluation.

### Evaluation of the results

The EQA scheme consisted of two consecutive rounds: the centers that failed the first round had the chance to register for a second round and were provided with another set of 10 samples. A board of assessors from AIOM and SIAPEC evaluated the results according to a predefined scoring system, in accordance with the European guidelines (Table 1) [13].

**Table 1:** Scoring system

Criteria	Marks
Correct genotype	2.00
Error in the nomenclature that might lead to misinterpretation of the results	1.5^a^
Genotype mispositioned or miscalled (partially correct diagnosis)	1
Test failure living no result on the sample	0.5
Incorrect genotype (false negative or false positive)	0

^a^Deduction applied only once

In order to pass the scheme, the AIOM-SIAPEC board established that both a score ≥18 and the absence of false positive or negative results are compulsory, since a serious genotyping error can adversely influence the identification of the correct therapeutic approach.

The participating laboratories received a report with the detailed results of each round. In particular, for each sample, the score and a comment to explain the errors were indicated.

## Results

### Selection of the samples for the EQA scheme

The EQA scheme was composed of two rounds, the second one open only to those centers which failed the first round. FFPE specimens with an adequate tumor cell content (≥50 %) were selected following analysis in three referral centers. Of the samples sent both for the first and the second round, 5 were wild-type for exons 2, 3 and 4 of *KRAS* and *NRAS* genes and for exon 15 of *BRAF* gene, while each of the other 5 samples had a mutation either in *KRAS* or in *NRAS* or in *BRAF* (Table 2).

**Table 2:** Mutational status of the samples selected for the first (A-label) and the second (B-label) round of the EQA

Sample ID	KRAS^a^	BRAF^a^	NRAS^a^
A1	WT	WT	WT
A2	WT	WT	WT
A3	WT	WT	WT
A4	WT	WT	WT
A5	WT	WT	WT
A6	WT	p.V600E (c.1799T > A) (27.6 %)	WT
A7	p.A146T (c.436G > A) (27.6 %)	WT	WT
A8	p.G12D (c.35G > A) (24.2 %)	WT	WT
A9	WT	WT	p.G12D (c.35G > A) (18.1 %)
A10	WT	WT	p.Q61K (c.181C > A) (74.6 %)
B1	WT	WT	WT
B2	WT	WT	WT
B3	WT	WT	WT
B4	WT	WT	WT
B5	WT	WT	WT
B6	p.G12D (c.35G > A) (27.7 %)	WT	WT
B7	p.A146T (c.436G > A) (62 %)	WT	WT
B8	WT	WT	p.Q61R (c.182A > G) (29.5 %)
B9	WT	WT	p.G12S (c.34G > A) (45.2 %)
B10	WT	p.V600E (c.1799T > A) (36.9 %)	WT

^a^In parentheses it is indicated the percentage of allele frequency evaluated by means of Ion Torrent

Overall, during the EQA 1200 samples were shipped and 1070 samples were effectively analysed (790 in the first and 280 in the second round). Testing was mandatory for exons 2, 3 and 4 of both *KRAS* and *NRAS* and for the *BRAF* V600E mutation.

### First round

Ninety centers registered on the web site http://www.rasquality.it, 4 of which from foreign countries. Two laboratories withdrew and 88 participated in the first round of the EQA. The participants were given 3 weeks to submit the results and 9 centers out of 88 were not able to fulfil this term. Thus, the results were obtained for 790 out of 880 shipped samples.

Analytical errors were observed in 41 of the 790 samples analysed (5.2 %), of which 17 (41.5 %) resulted to be false positives (3 laboratories with 2 false positives each), and 15 false negatives (36.6 %), with 2 centers having 2 false negatives each. In 8 cases (19.5 %) false negative and positive results occurred simultaneously in the same sample: for example, a sample that was mutated for *KRAS* and wild type for *NRAS* and *BRAF* but was incorrectly reported as *KRAS* and *NRAS* wild type and mutated for *BRAF*, was scored as false negative in *KRAS* and false positive in *BRAF*. In one sample (2.4 %) the genotype was mispositioned.

**Fig. 1:**
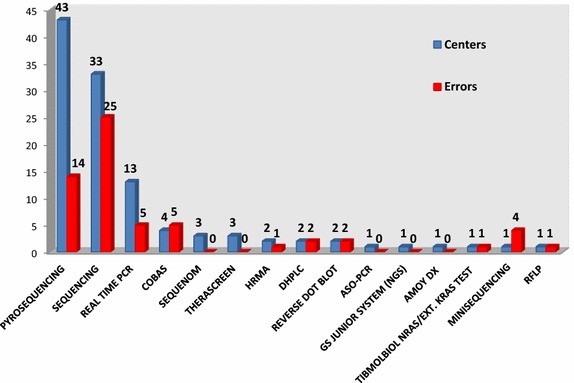
List of the methodologies used in the RAS EQA and relative errors. The *blue bar* indicates the number of centers that used the related method; the *red bar* indicates the absolute number of errors on the total of analyses performed with the indicated method

The most used methods were pyrosequencing and Sanger sequencing while real-time PCR was the third most used approach, with a relevant gap between the number of centers that used sequencing-based approaches or real-time PCR (Fig. 1). Given that each sample was analyzed for *KRAS*, *NRAS* and *BRAF*, the total number of tests performed by means of pyrosequencing or Sanger sequencing were 1068 and 831, respectively. The results of the analyses performed with these two approaches were compared by Pearson Chi Square test. A significantly higher incidence of errors was detected in those cases where Sanger sequencing was chosen, in particular, in 25 analyses out of 831 (3 %). In contrast, the percentage of error decreased when pyrosequencing (14 errors out of 1068; 1.3 %) was used (*p* value 0.01) (Fig. 1). Efficiency of the other methods could not be evaluated since they were underrepresented.

As shown in Table 3, the higher genotyping score was obtained for sample A8 carrying a *KRAS* p.G12D mutation, while the lowest was obtained for samples A7 (*KRAS* p.A146T) and A10 (*NRAS* p.Q61K).

**Table 3:** Score per case of the samples analysed in the first round

Sample number	Mutational status	Genotyping score^a^
A1	WT	1.95
A2	WT	1.95
A3	WT	1.92
A4	WT	1.85
A5	WT	1.97
A6	BRAF p.V600E	1.83
A7	KRAS p.A146T	1.56
A8	KRAS p.G12D	1.92
A9	NRAS p.G12D	1.81
A10	NRAS p.Q61K	1.67

^a^Not considering nomenclature errors

Twenty-two (27.8 %) laboratories of the 79 that eventually sent the results obtained the maximum score of 20 points. The score of 18 was obtained by 5 centers, but all of them made one genotyping error, thus failing the round. Only one center had a very poor performance, with a score of 9 points, but the laboratory retrospectively declared to have analysed only *KRAS* exon 2; the laboratory was automatically included in the second round.

Among the 88 centers that took part in the EQA, 56 (64 %) obtained a score >18 and passed the EQA. The remaining 32 laboratories were enrolled in the second round, including 3 foreign centers that did not pass the first round.

### Second round

In the second round, another set of 10 samples was sent to the 32 participating laboratories approximately 3 months after the completion of the first round (Table 2). Twenty-eight laboratories submitted the results within the 3-week deadline, for a total number of 280 results inserted on the website.

On the whole, 10 (3.5 %) errors were reported (false positives: 3, 30 %; false negatives: 2, 20 %, both false positive and negative results: 1, 10 %; mispositioned genotype: 4, 40 %). Among the 28 centers that submitted the results, 23 passed the second round, while 5 made at least one error. Of the 3 foreign laboratories that participated to the second round, only one had a score ≥18 and no major genotyping error. Overall, the laboratories improved their performance, with 20/32 centers (62.5 %) getting the maximum score and 23 laboratories (71.8 %) passing the round. Among the 5 centers that made errors, except for one laboratory that had not submitted the results within the deadline in the first round, the remaining 4 centers improved their performances, although obtaining a score <18.

**Fig. 2:**
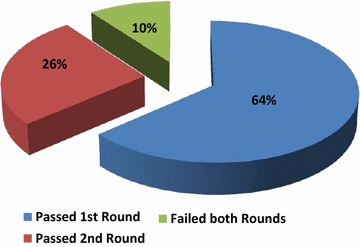
Results of the Italian external quality assessment for all-*RAS* and *BRAF* testing in colorectal carcinoma

Eventually, 79 of 88 participants obtained a score ≥18 in the first or in the second round and passed the RAS EQA scheme, including 2 foreign centers (Fig. 2). The list of the laboratories that passed the scheme is published on the web site of AIOM and SIAPEC (http://www.aiom.it; http://www.siapec.it).

### Clinical report evaluation

Reports were uploaded on the EQA website by all the participants; they were evaluated only on a qualitative level and no mark was given to them. The structure of the reports was extremely variable among the laboratories, with the exception of fixed criteria which were normally indicated such as patients name, date of birth and test result. Relevant information such as specification of mutations tested, percentage of neoplastic cells, characteristics of the methods used and interpretation of the results were at times or rarely indicated in the reports.

Significant variations were noticed in the genotype specification, since reference sequence was almost never declared and standardized Human Genome Variation Society nomenclature was incorrectly used to describe the mutations identified [14].

## Discussion

The rapid development and approval of new drugs that target molecular features of cancer cells is leading to a significant improvement of personalised medicine in oncology. Among the compounds available for the targeted therapy of CRC, cetuximab and panitumumab have been demonstrated to be active in *RAS* wild type patients. The AIOM and SIAPEC scientific board decided to introduce also *BRAF* mutational analysis in the RAS EQA, although not recommended in the EMA guidelines, also to fulfil the oncologist needs, since it can provide additional information on patient’s prognosis.

The RAS EQA was focused only on the genotyping analysis, and not on the pre-analytical phase, in order to reduce inter-laboratory variability due to the steps of dissections and evaluation of tumor cell content: thus it was possible to focus on the comparative assessment of the sensitivity and specificity of the methods used in the different laboratories [15].

As in the previous Italian EQA schemes [16, 17], the samples used for the tests were FFPE tissues, to ensure a stringent correlation with routine diagnostic activity, as in other European EQA [18, 19]. A total amount of 10 samples, suggested to be the adequate number of cases for proficiency testing in this field [13, 19], was used both for the first and the second round. However, in the Italian EQA schemes the same tissue samples are distributed to all participating centers, whereas groups of 10–15 laboratories on the basis of a regional distribution received different samples in the ESP EQA schemes [20]. In addition, EMQN uses as standards FFPE blocks containing cell lines. Therefore, the Italian schemes are the only to distribute the same tumor tissues to all participating laboratories, thus rendering possible a true inter-laboratory comparison among all participants with samples that resemble the routine clinical practice.

Nine centers did not meet the three-week deadline in the first round and four in the second round. No justification was provided by these centers. Because all-*RAS* testing was implemented shortly before the start of the EQA in most Italian centers, lack of experience in testing and problems of supply of reagents might have caused this phenomenon.

Although after the two rounds 79 centers out of 88 (89.8 %) passed the scheme, the error rate in the first round was worrying. Indeed, 36 % of the centers did not pass the first round, with 23 centers out of the 79 that submitted the results (29.1 %) having at least one false positive or false negative. These data are in line with the recently published results of the ESP RAS EQA showing a high rate of genotyping errors, although lower as compared to our results (3.6 vs 5.2 % in this study) [20]. In this respect, it must be emphasized that in the ESP EQA RAS 2014, full *RAS* and *BRAF* testing were not mandatory and only 49.3 % of the participants had completely implemented the new test requirements at the time of samples shipment [20]. Therefore, this is the first EQA to report data on full *RAS* and *BRAF* testing. Interestingly, the lowest average scores were located in exon 4 of *KRAS* and in exon 3 of *NRAS* gene, whereas the error rate for *NRAS* exon 2 was similar to *KRAS* exon 2. The number of errors for the codons recently added by the EMA was similar in the Italian and ESP EQAs. For example, the genotyping error rate for *NRAS* exon 3 were 16.5 and 15.4 % in the Italian and ESP EQA schemes, respectively. Because less than 50 % of the centers in the ESP scheme analysed all *RAS* exons, this might in part explain the difference in the overall error rate between the AIOM-SIAPEC and the ESP schemes. These data underline that introduction of new testing requirements might lead to a higher error rate if adequate training is not performed. We have also to recognize that the samples used in this EQA had a relatively high frequency of mutant alleles and they were not challenging, although they do represent the average sample type that is encountered in routine diagnostics in CRC.

The scoring system approved by AIOM and SIAPEC determines the failure of laboratories that commit one single genotype error. In fact, false positives prevent the patients from obtaining an active therapy, while false negatives cause the detrimental effects since they result in the administration of an inactive and potentially toxic compound to non-responder patients. Furthermore, treatment of *RAS* mutant CRC patients with anti-EGFR mAbs plus oxaliplatinum-based therapy has been shown to reduce patients’ survival [6]. The statistical analysis revealed a significantly better outcome of pyrosequencing compared to Sanger sequencing (Pearson Chi square p value: 0.01). This evidence might be related both to the ability of pyrosequencing to sequence short fragments, that allows an increased performance on FFPE samples, and to the higher sensitivity of this method compared to sequencing (5 vs 10–20 %) [16, 21]. Indeed, in this EQA the use of pyrosequencing surpassed the use of Sanger sequencing in routine diagnostic practice. Nevertheless, many laboratories that used Sanger sequencing had an excellent performance. This indicates that the sensitivity of the Sanger sequencing can vary among the different centers and it is also influenced by the single laboratory expertise. For these reasons, it would be appropriate for every laboratory to assess the limit of detection of the technique they currently use. The different sensitivity might account for the relatively high number of false negative results but opens the question of the number of false positive that were higher than false negatives in both rounds (I round: 17 vs 15; II round: 3 vs 2). Apart from potential contamination of the sample, a possible explanation is represented by potential artefacts introduced by PCR or sequencing cycle, especially when a low input DNA is used for amplification. In this regard, a high rate of false positive has also been found in the AIOM-SIAPEC EQA for *EGFR* testing that also showed a higher error rate for laboratories using Sanger sequencing as compared to those employing pyrosequencing or Real Time PCR [17]. Overall, these data suggest that Sanger sequencing should be adopted by centers with high experience in molecular biology because it requires an accurate standardization of the workflow.

Once again the importance of having two rounds within the same year was confirmed, since the majority of laboratories which had the chance to repeat the analyses, actually enhanced their performances, and surprisingly a high percentage of centers (72 %) passes the second round, obtaining in most cases the 100 % of correct answers. The fact that approximately 3 months passed between the two rounds might also account for the significant improvement in performance of the majority of centers. However, 9 centers did not pass the EQA and no consequence of poor performance is foreseen in the Italian health system. Nevertheless, AIOM and SIAPEC publish the list of the centers that did pass the EQA, thus allowing medical oncologists and patients to choose laboratories that successfully participated in the scheme. In addition, the Italian scientific societies offer training for centers with poor performance in order to help them to improve their quality.

Review of clinical reports showed a number of issues, although they received only a qualitative evaluation. In the first place, the majority of laboratories included a number of essential elements, namely patient ID, date of birth, method applied and results found. On the other hand, several critical details were missing, such as type of specimen used, limit of detection of the methods applied, reference sequence and clinical interpretation, leading to ambiguous and unclear reports. Information regarding for example the sensitivity and method limits might be crucial for the clinical interpretation of the obtained results, since a test that might not be able to detect all the relevant mutations or that is not appropriately sensitive for a challenging specimen may mislead treatment decision.

In general, the results of the II Italian RAS EQA scheme indicate that the mutational analyses are performed with good quality in many Italian centers, although a lack in uniformity in reporting the results was pointed out. The high number of centers failing the first round underline the fundamental role in continued education covered by EQA schemes, which aim at identifying errors in methodologies and ensuring an adequate quality of molecular testing. Moreover, the writing of guidelines to implement the uniformity of analyses and of reporting both genotyping data and results is essential to increase high-quality performance.

Finally, the introduction of new biomarkers in the identification of the optimal pharmacological approach to cancer determines the continuous development of testing requirement. In the next future personalized medicine will be based on the availability of new compounds and the combination or the sequential use of therapies will be implemented to overcome resistance mechanisms. Inevitably, not only the number of biomarkers to test but also of patients requiring molecular analyses will increase, further enhancing the need for reliable and constantly up-to-date testing services.

## Conclusions

Our study has highlighted the importance of external quality assessment in molecular pathology to ensure that patients receive adequate diagnostic test for their disease. The high error rate of the first round emphasizes the need for scientific societies, regulatory authorities, manufacturers of diagnostic kits and dedicated professionals of the field to work together to improve the overall quality and accelerate the standardization of new tests necessary for patients with cancer.
